# In-Shoe Pressure Measurements in Diabetic Footwear Practice: Success Rate and Facilitators of and Barriers to Implementation

**DOI:** 10.3390/s24061795

**Published:** 2024-03-11

**Authors:** Jennefer B. J. Zwaferink, Frans Nollet, Sicco A. Bus

**Affiliations:** Department of Rehabilitation Medicine, Amsterdam UMC, University of Amsterdam, Amsterdam Movement Sciences, Meibergdreef 9, 1105 AZ Amsterdam, The Netherlands; j.b.j.pouwels@saxion.nl (J.B.J.Z.); f.nollet@amsterdamumc.nl (F.N.)

**Keywords:** diabetic foot, pressure measurement, custom-made footwear, implementation

## Abstract

We aimed to assess the success rate and facilitators of and the barriers to the implementation of in-shoe plantar pressure measurements in footwear practice for people with diabetes at high risk of foot ulceration. Eleven Dutch footwear practices were partly supported in purchasing a pressure measurement system. Over a 2.5-year period, trained shoe technicians evaluated 1030 people with diabetes (range: 13 to 156 across practices). The implementation success and associated facilitators and barriers were evaluated quantitatively using completed measurement forms and pressure measurement data obtained during four monitoring sessions and qualitatively through semi-structured interviews with technicians. Across the 11 practices, the primary target group (people with diabetes and a healed plantar foot ulcer) represented 25–90% of all the patients measured. The results showed that three practices were successful, five moderately successful, and three not successful. The facilitators included support by the company management board, collaboration with a prescribing physician, measurement sessions separate from the outpatient clinic, and a (dedicated) shoe technician experiencing a learning effect. The barriers included investment costs, usability aspects, and limited awareness among shoe technicians. In-shoe plantar pressure measurements can be implemented to a moderate to large degree in diabetic footwear practice. The barriers to and facilitators of implementation are organizational, logistical, financial, or technical, and the barriers are modifiable, supporting future implementation.

## 1. Introduction

Foot ulceration is a common complication of diabetes mellitus, with an annual incidence of about 2% in the general diabetes population [1]. After a healed ulcer, 40% of the people have a recurrent ulcer within one year and 60% within three years [2]. Apart from this history of ulceration, the important ulcer risk factors include peripheral neuropathy, peripheral artery disease, foot deformity, amputation, and end-stage renal disease [2,3,4,5]. Elevated peak plantar pressure during weight-bearing activity is also an important risk factor for ulceration and its recurrence [2,3,6]. This follows the most common mechanical pathway of foot ulceration, where motor neuropathy and foot deformity lead to biomechanical abnormalities, callus formation from repetitive stress, and eventually skin breakdown [1,2]. To heal and prevent ulcers on the plantar foot surface, the mitigation of this stress is one of the mainstays of treatment in diabetic foot disease [7].

To help prevent plantar foot ulcer recurrence, custom-made footwear is commonly prescribed to high-risk people with diabetes; this footwear mainly aims to reduce plantar peak pressure inside the shoe [8]. This can be achieved by specific design features of the shoe, including a rocker outsole, outsole stiffness, custom-made insole, and different insole elements, such as a metatarsal pad or bar and top cover [9,10,11,12,13,14,15,16,17,18,19]. In-shoe plantar pressure measurement is a tool with which the pressure-relieving capacity of such custom-made footwear and design elements can be evaluated and is widely used in scientific research [5,20,21,22,23]. Additionally, in-shoe plantar pressure measurement has been shown to be valuable as a diagnostic method to identify high-pressure regions and to then guide the modification of footwear with the goal of reducing peak pressure at these locations after footwear delivery [16,20]. This application was first described by Mueller et al. [11] and later elaborated on and protocolized by Bus et al. [21] and Waaijman et al. [20]. Randomized controlled trials show that the use of plantar pressure analysis as a guidance tool to improve pressure relief by footwear significantly reduces the incidence of foot ulcer recurrence in people with diabetes, when the footwear is worn as recommended [24,25,26]. As a result, international guidelines of the International Working Group on the Diabetic Foot (IWGDF) recommend the use of custom-made footwear with a demonstrated plantar pressure-relieving effect for the prevention of foot ulcer recurrence in people with diabetes who are at high risk of ulceration [8].

Despite the fact that the primary aim of custom-made footwear is to distribute plantar pressure and the available scientific evidence and clinical recommendations for using pressure-driven custom-made footwear designs, in-shoe plantar pressure measurement is not yet widely used in diabetic footwear practice. This may be because custom-made footwear prescription is only slowly changing from a traditional experience- and skills-based approach to a more data- and scientifically driven approach. Furthermore, such measurements require investments in equipment, the training of personnel, and the time to conduct the measurements. When such investments are not (yet) part of reimbursed care, the use of in-shoe plantar pressure measurement relies on the willingness of those prescribing and/or manufacturing footwear to pay for this service. In many countries, including the Netherlands, custom-made footwear is prescribed by physicians and designed and manufactured by technicians (also called pedorthists) of footwear companies that are affiliated with or contracted to outpatient foot clinics within hospitals. Many of these physicians and shoe technicians see the benefit of using in-shoe plantar pressure analysis for evaluating and improving custom-made footwear and are willing to invest in this service, pending contracts for the reimbursement of the service. However, to date, there are no studies that have investigated the feasibility of implementing in-shoe plantar pressure measurements in clinical footwear practice. 

Given the clinical benefit of plantar pressure measurement, information on how successfully it can be implemented and what the facilitators of and barriers to such implementation are would help to progress the use of evidence-based pressure-driven footwear design and evaluation in footwear practice. The aim of this study was therefore to assess the success rate and facilitators of and the barriers to using in-shoe plantar pressure measurements in footwear practice for evaluating the custom-made footwear of people with diabetes who are at high risk of foot ulceration.

## 2. Materials and Methods

### 2.1. Study Design

This was a prospective observational study monitoring the use of in-shoe pressure measurements in footwear practice over a 2.5-year period. To assess the implementation, the study collected quantitative data through reported measurements and qualitative data through semi-quantitative interviews.

### 2.2. Participating Companies and Conditions

In the Netherlands, custom-made footwear for people with diabetes is prescribed by physicians and designed and manufactured by shoe technicians working at orthopedic footwear companies that are contracted to outpatient foot clinics within hospitals. Orthopedic footwear companies often have regional or even national coverage and are contracted to more than one hospital. Furthermore, most footwear companies use a workshop within the hospital to modify the shoe when needed and have multiple branches outside of the hospital where patients can have their prescribed footwear fitted, evaluated, and collected.

All the footwear companies that were members of the Dutch branch organization for orthopedic shoe technicians (NVOS-Orthobanda) were contacted by email to inform them about this implementation project, the possibility to participate, and the seminars that would be organized to discuss the project; the requirements for participation were communicated, and the companies were asked to express their willingness to participate. Three seminars were held at locations spread throughout the Netherlands; 42 shoe technicians and managers from 20 footwear companies participated. 

The participants in the project had to meet the following preset requirements: (1) a sufficient number of eligible patients for in-shoe pressure measurements to justify support for the purchase and use of an in-shoe pressure measurement system, as judged by the investigators; (2) willingness to purchase, with 50% support in costs through the project grant, a Novel Pedar-X in-shoe pressure measurement system; (3) willingness to adjust business operations to enable measurements; (4) willingness to share collected data anonymously with the investigating team; and (5) the availability of sufficient space to carry out the measurements and an adjacent workshop for immediate footwear adjustments if needed. 

The companies could participate as a stand-alone footwear practice, in collaboration with other companies sharing one pressure measurement system, or by outsourcing the pressure measurements to a facility such as a gait laboratory where the pressure measurement equipment is used. After willingness to participate was expressed, the eligibility to participate was assessed based on an intake interview. This project was funded by a grant from the Development Fund for Orthopedic Shoe Companies (OFOM) in the Netherlands.

### 2.3. Procedures

Prior to the start of the project, the participants were trained by Novel GmbH in the fundamental and technical aspects of plantar pressure measurement and in using the in-shoe pressure measurement system. After the training, the participants were provided with a Pedar-X measurement protocol, which was a simplified version of the protocol used in the gait laboratory of the Amsterdam UMC; the protocol outlined the required steps to conduct a valid and reliable in-shoe pressure measurement with the Pedar-X measurement system. Additionally, the participants were provided with the Amsterdam UMC protocol for the use of in-shoe pressure measurements for footwear evaluation and modification that aims to improve the pressure-relieving capacity of custom-made footwear. Via a flow diagram, this protocol describes the conditions under which shoe modifications should be made and includes two matrices that illustrate the pressure-relieving effect of the (combination of) shoe modifications on a specific region, based on previously published data and schemes from the DIAFOS research project [9,20].

For the duration of the project, the participants were instructed to evaluate every person with diabetes mellitus who had a history of foot ulceration and was provided with some form of custom-made footwear. 

In-shoe pressures were measured using the Pedar-X system (Novel, Munich, Germany) during overground walking at a comfortable speed. The Pedar-X system consists of 2 mm thick flexible insoles with 99 capacitance-based sensors that each measure at a 50 Hz sample rate. The system works by inserting the measurement insoles in the shoes and placing them on top of the insoles. A data cable connects the insole to a data logger that is worn around the waist of the subject and that transmits data in real-time via a Bluetooth connection to the computer. A minimum of 12 midgait steps per foot are measured to obtain valid and reliable data for the participant [20]. If the plantar peak pressure at the toes, forefoot, or midfoot was >200 kPa, according to the protocol, the shoe technician was instructed to modify the footwear and reassess the in-shoe plantar pressures until the peak pressures were below an absolute level of 200 kPa or reduced by 25% compared to the baseline assessment [20]. The shoe technician selected the footwear modification and used the evaluation protocol for guidance ad libitum. Multiple modifications could be made at once, and according to protocol, a maximum of two rounds of adjustments and subsequent pressure evaluations were conducted. After each patient measurement session, the participants completed a form in which they entered demographic, measured pressure, and shoe technical data. The participants were instructed to re-evaluate the in-shoe pressures six months after footwear delivery, using the same protocol.

The participants were instructed to have the Pedar-X measurement system calibrated twice a year. The calibration device of the gait laboratory at Amsterdam UMC was used for this purpose, and the calibration was conducted by gait lab personnel, against payment of a fee commensurate with their time investment. 

The primary target group comprised people with diabetes who had a history of plantar foot ulceration and for whom in-shoe plantar measurements are an evidence-based evaluation procedure [24]. Other patient groups could additionally be measured by the participant and could include people with diabetes and a foot ulcer or people with diabetes without a foot ulcer history but with an indication of pressure evaluation based on pressure-related issues with the footwear or present signs of increased pressure such as calluses or red spots. Prior to the start of the measurements, all the patients measured provided written informed consent to have their collected data used for scientific purposes when needed.

### 2.4. Monitoring and Data Collection

The project had a duration of 2.5 years. Throughout the project, the investigator (JBJZ) conducted four monitoring sessions per participant: three during the project and one final visit after the project’s end date. These sessions aimed to collect both quantitative and qualitative data to assess implementation success and to assess the facilitators of and barriers to this implementation. The qualitative data were collected through semi-structured interviews with the person responsible for the measurements at each center. During these interviews, a standard set of closed and open-ended questions were asked, and the answers were synchronously documented in Microsoft Word during the interviews by the investigator (JBZ). The obtained organizational data included (a) the start date, (b) the time investment per patient, (c) the number of footwear branch locations where measurements were conducted, (d) the setting for the measurements, either during an outpatient clinic or in separate sessions, (e) the involvement of a physician, (f) the re-evaluation of in-shoe pressures after 6 months, and (g) the calibration of the measurement system. Additionally, the participants were asked to provide insights into the facilitators of and barriers to the use of the in-shoe pressure measurement system and the impact of conducting these measurements on their professional development (i.e., learning effect and efficiency of footwear adjustments). 

The quantitative data were collected through the completed measurement forms and in-shoe pressure data collected per patient. The organizational data included the number of unique patient measurements conducted. The footwear data included (a) type of prescription, (b) footwear design elements, (c) type of modifications, and (d) in-shoe pressure data collected. The patient data included (a) demographic parameters, (b) disease-related parameters, and (c) clinical outcomes over time, if available. 

The implementation of in-shoe pressure measurements in footwear practice was classified as fully successful, moderately successful, or not successful. The implementation was considered fully successful when a participant met each of the following four criteria: (1) a minimum of 50 unique patient measurements a year were conducted; (2) more than 60% of the measurements were conducted in the primary target group (i.e., people with diabetes and a healed foot ulcer); (3) in-shoe pressures were re-evaluated every six months; and (4) the measurement system was calibrated at least once a year. The implementation was considered moderately successful when a minimum of 35 unique patient measurements a year were conducted and at least one of the above from criteria 2 to 4 was met. The implementation was considered unsuccessful when none of the four criteria were met or when a participant discontinued the measurements before the end of the study period of 2.5 years.

The footwear design data, in-shoe pressure data, and clinical outcome data were not analyzed for the current study since these data are beyond the scope of this article. 

### 2.5. Data Analysis

The organizational data for the evaluation of implementation success were collated per participant in a table. All the evaluation parameters were analyzed using descriptive statistical analyses in SPSS for Windows (IBM SPSS Statistics version 22, Armonk, NY, USA). All the semi-structured interviews were documented by the first author (JBJZ) and analyzed based on the method of Braun and Clarke [27]. All the statements by those interviewed were coded and grouped into categories to identify the themes regarding facilitators and barriers.

## 3. Results

The organizational data per participant are presented in Table 1. In total, 17 footwear companies participated in this project. Seven of these companies jointly participated as a footwear practice and purchased and shared one in-shoe pressure measurement system. The remaining ten companies participated independently, and each company purchased an in-shoe pressure measurement system. As a result, the analysis was conducted on 11 footwear practices, and they are named as such in the remainder of the manuscript. The duration of the follow-up varied across practices since not all of them started collecting data at the start of the project for organizational reasons. Six practices started between September and November 2015, three between January and April 2016, and two between September and October 2016. 

### 3.1. Number of Measurements

In-shoe plantar pressures were measured in a total of 1030 people with diabetes, ranging from 13 to 156 across the practices during the project (Table 1). A total 525 measurements (51%) were in the primary target group, and 505 measurements (49%) were in people with diabetes and a foot ulcer or in people with diabetes with no ulcer history but with an indication for pressure evaluation based on pressure-related issues with the footwear or present signs of increased pressure such as calluses or red spots. Three practices measured in-shoe pressures in more than seventy people per year, two in more than fifty people, and three in more than forty people. Two practices measured less than twenty people per year.

### 3.2. Primary Target Group 

The percentage of measurements conducted in the primary target group (i.e., people with diabetes and a healed ulcer) varied between 25% and 90% of the total measured groups across the practices. Two practices had more than 75% of the measurements in the primary target group, four practices between 50% and 75%, and five practices below 50%. The latter five practices conducted the majority of the in-shoe pressure measurements in people with diabetes and a foot ulcer or in those indicated by a pre-ulcer presence or complaint about the footwear.

### 3.3. Re-Evaluation after 6 Months and Calibration of the Measurement System

Three practices reported that they re-evaluated in-shoe plantar pressures in their patients every six months, as instructed; the other eight did not. Six of the practices had their measurement system calibrated once a year, and five did not. None of the practices calibrated their measurement system twice a year, as was instructed.

### 3.4. Measurement Facilities, Sessions, and Time Slots

The number of facilities (i.e., company branch locations or outpatient foot clinics) where patients were measured for in-shoe pressure varied from one to six across the practices (Table 1). Seven practices organized a standalone session separate from the outpatient clinic to conduct the in-shoe pressure measurements and footwear modifications. The other four conducted measurements during the outpatient clinic. Eight practices planned 60-min time slots to evaluate one patient for pressure measurement and footwear modification, one practice planned 45-min time slots and two practices planned 30- to 45-min time slots.

### 3.5. Involvement of a Physician

Seven practices had direct collaboration with a physician in an outpatient diabetic foot clinic, where custom-made footwear was prescribed and pressure measurements were ordered. Four practices did not have a physician involved in the procedure. 

### 3.6. Technical Aspects 

Some practices experienced technical issues with the measuring system, such as a broken connection cable between the insole and the device and malfunctioning sensors of an insole. In addition, all the practices experienced difficulties measuring people with larger or smaller feet than the available sizes of the measuring insoles and with heavily abnormal foot shapes, such as a Charcot foot or an amputation.

### 3.7. Implementation Success

Based on the criteria for successful implementation, the implementation of in-shoe pressure measurements was considered fully successful in two practices and moderately successful in six practices (Table 1). In three practices, the implementation was considered unsuccessful; these practices stopped conducting in-shoe pressure measurements before the end of the project, and they did not intend to restart measurements after the project.

### 3.8. Facilitators and Barriers (Based on Semi-Structured Interviews)

Four themes regarding facilitators were identified: (1) learning effect; (2) support from the management board; (3) patient experience; and (4) collaboration with a prescribing physician. Also, four themes regarding barriers were identified: (1) technical challenges; (2) investment of costs and time; (3) referral to other shoe technicians; and (4) logistical challenges. Regarding the first theme among the facilitators, a positive learning effect among shoe technicians/measurers was reported by 8 of the 11 practices, and a reduction in the required number of footwear modifications over time was also reported by 8 practices. Two practices mentioned that this learning effect translated into a reduction in the time required for a measurement session, and three practices mentioned changes in footwear design based on gained experience in pressure measurement outcomes. Regarding the second theme, in three practices the board reported that the in-shoe pressure measurements contributed to an improvement of quality in footwear care, and in two practices, it was reported that in-shoe pressure measurements could be used to distinguish them from competitors. Regarding the third theme, three practices reported that the evaluated patients were positive regarding the in-shoe pressure measurements and that they appreciated the attention and time devoted to them. Additionally, enhanced patient awareness of the importance of appropriate footwear due to the visualization of pressure outcomes was reported by two practices. Regarding the fourth theme, two practices reported that they used the pressure measurements as evidence in discussions with the physician in cases where there was debate about whether a problem was footwear-related or related to the non-adherence of the user. 

Among the barriers, within the first theme, 10 of 11 practices highlighted the complexity and friendliness of using the in-shoe pressure measurement system. System vulnerabilities, including Bluetooth connection problems and defects in sensors, cables, insoles, or batteries were reported by eight practices. Furthermore, four practices reported constraints related to the number and available sizes of measuring insoles and difficulties in measuring heavily deformed feet (*n* = 4). Regarding the second theme, five practices reported that the pressure measurement and footwear adjustment process was time-consuming, and four reported that the investment costs were high. Regarding the third theme, six practices reported having a limited awareness of the referral patients for a pressure measurement session, and four reported a resistance due to the concern that their manufactured shoes were being evaluated and (sometimes) adjusted by colleagues. Regarding the fourth theme, two practices reported logistical difficulties with conducting measurements with one system at multiple branch locations and with the availability of the system due to its rotation between branch locations. Additionally, the scheduling of patients who had been referred by other branch locations was reported as challenging by these practices.

## 4. Discussion

This is the first study that investigated the success and facilitators of and barriers to the implementation of in-shoe plantar pressure measurements in footwear practice for people with diabetic foot disease. The implementation of in-shoe pressure measurements was considered a full success or a moderate success in 8 of 11 footwear practices and as unsuccessful in the other 3. Several barriers to and facilitators of successful implementation are present and are discussed below. 

The in-shoe plantar pressures were measured during the 2.5-year project duration in 1030 individuals with diabetes, with a large range of 13 to 156 in the number of patients measured per practice. The low numbers in this range, i.e., those up to a total of 100 patients seen in 6 of 11 practices, may be attributed to practices overestimating the number of eligible patients for in-shoe pressure measurements. Additionally, a low awareness (or resistance) of shoe technicians regarding the measurement of patients may explain this outcome. Some practices did not measure all the eligible patients due to logistical challenges, such as the sharing of the system between branch locations of the same company or the scheduling of footwear deliveries at moments or locations where the system or a technician was not available for measuring the in-shoe plantar pressure. 

These findings indicate that only one to a few measurements per week were conducted with one measurement system, meaning that the system remained idle for most of the week. The efficiency of use was therefore low, giving a limited return on purchase, maintenance, and training costs. Furthermore, there is a risk of not building up sufficient experience in measuring in-shoe plantar pressures, particularly when more than one technician per practice is involved in conducting the measurements. A possible solution to this could be to setup a scheme whereby the measurement system is used within more branch locations of the company so that more people are measured. Another option may be to assign one or two technicians within the company to be responsible for the measurements, who then rotate between the different branch locations. Yet another option could be to refer patients to a geographically centrally located branch of the company to have their in-shoe pressures measured. This last option has the disadvantage that patients may need to travel a relatively long distance and that if any footwear modifications are necessary, they will not be conducted by the patient’s own shoe technician. Creative solutions are needed here to increase the efficiency of use of the in-shoe pressure measurement system.

The percentage of measurements conducted in the primary target group of people with a healed plantar foot ulcer ranged substantially across practices between 25% and 90% and was on average 51%. Five practices mostly measured people with diabetes who had a foot ulcer or those who had a pre-ulcer present or had a complaint about their footwear. Although in-shoe plantar pressure measurements in these groups may be highly valuable, this is not evidence-based. Additionally, while a peak pressure target level of 200 kPa is used as an evidence-based threshold for ulcer prevention in the primary target group [21,28], such a level may not apply to other groups measured. In patients with a foot ulcer, for example, a lower target pressure threshold is likely to apply. This awareness appears to be insufficiently present among shoe technicians and prescribing physicians.

Only 3 of the 11 footwear practices reassessed in-shoe plantar pressures after 6 months; most practices only measured at footwear delivery. As a result, changes in the pressure-relieving properties of the footwear over time remained unnoticed. The DIAFOS study showed that the pressure-relieving properties of the footwear change over time, even within 3 months, which is likely due to the wear of the materials, emphasizing the need for timely re-evaluation of in-shoe plantar pressure [24]. As a possible explanation, most practices reported that the re-evaluation of in-shoe plantar pressures is not integrated into their routine practice, where they usually only see the patient return for monitoring of their footwear when the patient develops a foot problem or has a complaint about the footwear. Implementing a standard procedure of scheduling re-evaluations over the lifetime of the prescribed footwear at footwear delivery may help to improve the re-evaluations. Additionally, re-evaluating the footwear every six months requires extra time and costs, which may even be doubled or tripled compared to those of the single measurement. Shoe companies seem reluctant to invest in such measurements over time if they do not receive compensation through the reimbursement system, despite the fact that a positive effect of such reassessments is that the efficiency of using the system is improved. Having such reimbursement implemented will also improve the re-evaluations of the footwear.

A technical aspect that received very little attention from the footwear practices is the calibration of the measuring system. The Amsterdam UMC has a calibration device for the Pedar-X system, and the footwear practices had the option to have their measurement system checked and calibrated against payment of a fee. Despite the recommendation in the project to calibrate the system every six months, none of the practices followed this recommendation, and only six practices calibrated their systems once per year. The other five did not calibrate their systems at all. A potential explanation may be that the practices do not perceive the necessity to calibrate such a system, as they may not be aware of the importance of calibration, compared for example with researchers or lab personnel. This may also explain why none of the practices purchased their own calibration system. Additionally, the necessity to send their Pedar-X system to Amsterdam UMC for calibration, causing temporary unavailability and a fee payment, may have made the practices reluctant to have the system calibrated. Such calibration is not specific to the Pedar-X system used in the project, as all measurement systems must be calibrated at some point to ensure accurate and reliable outcomes. Further stressing to users that calibration will help to maintain accurate outcomes is probably needed to improve calibration. Additionally, the participants purchasing and using their own calibration systems or automatic reminders to conduct a pressure system calibration every several months may help.

Most of the practices scheduled sessions for in-shoe pressure evaluation and footwear modification that were separate from the often busy outpatient foot clinics. An important finding of this project is that almost all of the successful and moderately successful practices used separate measurement sessions; the ones that were not successful all performed the measurements in outpatient foot clinics. While such a setup probably requires patients to make an extra visit for footwear evaluation, the results suggest that this is necessary for successful implementation. 

Furthermore, all but one of the successful or moderately successful practices collaborated directly with a prescribing physician in the clinic that they were affiliated with, which likely facilitated implementation. Still, in most cases, the shoe technician requested and scheduled the pressure measurements, not the prescribing physician, who ordered the pressure measurements in only a few cases. Given the clinical implications and the responsibilities involved with evaluating and improving footwear, it is desirable that in-shoe plantar pressure measurements are performed in close collaboration between the physician and technician.

### 4.1. Facilitators of and Barriers to Implementation

Based on the analysis of the quantitative findings, implementation seems facilitated by the use of sessions for in-shoe pressure measurement and footwear modification, which are separate from the normally busy outpatient clinics, are preferably at a fixed time during the week, and use 45–60 min time slots per patient. Having multiple patients scheduled per session facilitates efficiency in the use of the system. Using the measurement system across branch locations, such as by rotating the system or by rotating technicians between locations, helps to measure more eligible patients and to increase efficiency. 

The quantitative and qualitative data seem to show that close collaboration and shared decision making between the prescribing physician and the shoe technician also facilitates implementation and helps to increase the percentage of eligible patients being measured. 

The support of the company’s management board for the use of such a service is also an important facilitator.

The qualitative analysis shows that a positive learning effect and positive patient experience seem to facilitate implementation. Improved skills in measuring and adjusting footwear over time increases efficiency and reduces the time required for a measurement session. This may enhance the feasibility of using in-shoe pressure measurements in footwear practice and the efficacy of the footwear provided. 

A barrier to implementation is the usability of the pressure measurement system perceived by the involved shoe technicians. These pressure measurement systems were originally developed primarily for research purposes and used by highly qualified and trained scientists and lab personnel. Their use by less trained shoe technicians may require an adaptation of the system. The reported technical issues, including system malfunctions and constraints related to the number and available sizes of measuring soles, limit the number of people that can be measured (in the time available). Measuring people with abnormal foot shape due to Charcot deformity or amputation may require further investments in measurement insoles for these cases. 

Another barrier to implementation is the required investments in costs and time for conducting the in-shoe pressure measurements. A solution to this barrier would be to have the costs for the service reimbursed or otherwise paid for. A cost-effectiveness analysis of the outcomes of the DIAFOS trial shows that custom-made footwear that is evaluated and, if needed, modified at intervals of 3 months using in-shoe plantar pressure analysis is more cost-effective than the usual care [29]. Such findings can hopefully help to engage the users and potential funders of this service in discussions regarding reimbursement. 

As a barrier, the limited awareness or resistance of shoe technicians regarding measuring patients or referring patients for an in-shoe pressure measurement indicates the importance of finding a solution for the integration of the service into the standard workflow of shoe technicians.

### 4.2. Study Limitations

Firstly, the implementation was partially facilitated by compensating half of the purchase costs for the pressure measurement system through the study grant. While this may reflect how footwear companies see this service. i.e., as a reimbursed option, it may affect participation and motivation and, consequently, the implementation outcomes. A second limitation is the limited number of practices in two of the three participating setups, i.e., the sharing of a system between companies in one footwear practice and the outsourcing of the measurements, e.g., to a gait laboratory. For a better comparison between setups, more practices per setup would be needed. A third limitation is the inherent subjectivity present in much of the data collection, such as in the classifying of implementation success and the experiences from footwear practices. Lastly, the outcomes may be specific to the Dutch context and may have limited generalizability to footwear settings in other countries, where the system for providing footwear for people with diabetes may be different regarding healthcare policies, reimbursement, available resources, and the organization of foot care and footwear services.

## 5. Conclusions

In-shoe plantar pressure measurements can be implemented to a moderate to full degree in footwear practice for people with diabetes. Implementation success largely depends on organizational/logistical, financial, and technical factors, most of which are modifiable. While implementation was not fully successful across practices, the modifiable barriers highlight the potential for the implementation of this service for the evaluation of custom-made footwear for people with diabetes.

## Figures and Tables

**Table 1:** Organizational data per participant.

Participant (Footwear Practice)	Start Month/Year	Nr. of Branch Locations for Measurement	Measurement Sessions Separate from Clinic	Involvement of Physician	Nr. of People Conducting Measurement + Profession	Footwear Modified By Same Persons Who Measures?	Time Planned per Measurement (min)	Most Measured Patient Group	Total nr. of Unique Patient Measurements	Nr. of Unique Patient Measurements Per Year	% Measurements in Primary Target Group	Re-Evaluation of Footwear after 6 Months	System Calibration	Learning Effect	Plans for Continuation after the Project	Successful Implementation?
**1**	09/2015	6	Yes	Yes, 3/6	2 OST	Yes	60	PTG	156	78	60%	Yes	Yes	Yes	Optimize + expand	Fully
**2**	09/2015	2	Yes	Yes	2 OST	Yes	45	PTG	123	55	65%	Yes	Yes	Yes	Optimize	Fully
**3**	10/2015	3	Yes	No	2 OST	Yes	30–45	PTG	125	58	80%	No	Yes	Yes	Continue as is now	Moderately
**4**	03/2016	2	Yes	Yes, 1/2	3 POD	No, 3 OST	60	ULC + IND	147	84	35%	Yes	No	Yes	Optimize	Moderately
**5**	04/2016	1	Yes	Yes	3 LAB	No, 2 OST	30–45	ULC + IND	118	71	25%	No	Yes	Yes	Optimize	Moderately
**6**	10/2015	5	Yes	Yes, 2/5	2 OSTst	Yes	60	PTG	98	45	65%	No	No	Yes	Optimize + expand	Moderately
**7**	09/2016	4	Yes	No	4 OSTst	No, 4 OST	60	ULC	59	47	25%	No	Partly	Yes	Optimize + expand	Moderately
**8**	11/2015	2	No	Yes	2 OST	Yes	60	ULC + IND	77	37	35%	No	Yes	Yes	Expand	Moderately
**9**	10/2015	4	No	Yes, 1/4	4 OSTst	Yes	60	ULC + IND	86	40	35%	No	No	Yes	Unsure	No
**10**	01/2016	1	No	No	1 OST	Yes	60	PTG	28	15	90%	No	No	-	No	No
**11**	10/2016	1	No	No	1 OST	Yes	60	PTG	13	11	75%	No	No	-	No	No

Data are N, or otherwise indicated. OST: orthopedic shoe technician; POD: podiatrist; OSTst: OST student; LAB: lab technician. PTG: primary target group; ULC: ulcer group; IND: on indication. Pressure measurements were conducted ‘on indication’ when users had a complaint about the footwear or when there were signs of increased pressure such as red spots or calluses. In the column “Involvement of Physician”, the fraction mentioned is the number of branch locations of the total in which a physician was involved. “Learning effect” indicates whether technicians reported having a learning curve in optimizing the pressure-reducing properties of the footwear by conducting measurements. “Optimize” indicates that the participant will continue with conducting measurements and aims to make further improvements to the service, such as expanding the number of people measured, increasing the number of measurements within the primary group, or implementing follow-up measurements after six months. “Expand” indicates that the participant intends to extend the in-shoe pressure measurements to additional branch locations of the company. The green cells indicate which of the four criteria for implementation success were met. The last column indicates whether the implementation was successful (green cells), moderately successful (oranges cells) or not successful (red cells).

## Data Availability

The data of this study are all contained within the manuscript and also available upon request to the corresponding author.

## References

[1] Armstrong D.G., Tan T.-W., Boulton A.J.M., Bus S.A. (2023). Diabetic Foot Ulcers: A Review. JAMA.

[2] Armstrong D.G., Boulton A.J.M., Bus S.A. (2017). Diabetic Foot Ulcers and Their Recurrence. N. Engl. J. Med..

[3] Monteiro-Soares M., Boyko E.J., Ribeiro J., Ribeiro I., Dinis-Ribeiro M. (2012). Predictive factors for diabetic foot ulceration: A systematic review. Diabetes Metab. Res. Rev..

[4] Crawford F., Cezard G., Chappell F.M., Murray G.D., Price J.F., Sheikh A., Simpson C.R., Stansby G.P., Young M.J. (2015). A systematic review and individual patient data meta-analysis of prognostic factors for foot ulceration in people with diabetes: The international research collaboration for the prediction of diabetic foot ulcerations (PODUS). Health Technol. Assess..

[5] Van Netten J.J., Raspovic A., Lavery L.A., Monteiro-Soares M., Paton J., Rasmussen A., Sacco I.C.N., Bus S.A. (2023). Prevention of foot ulcers in persons with diabetes at risk of ulceration: A systematic review and meta-analysis. Diabetes Metab. Res. Rev..

[6] Waaijman R., De Haart M., Arts M.L.J., Wever D., Verlouw A.J.W.E., Nollet F., Bus S.A. (2014). Risk Factors for Plantar Foot Ulcer Recurrence in Neuropathic Diabetic Patients. Diabetes Care.

[7] Lazzarini P.A., Crews R.T., Van Netten J.J., Bus S.A., Fernando M.E., Chadwick P.J., Najafi B. (2019). Measuring Plantar Tissue Stress in People with Diabetic Peripheral Neuropathy: A Critical Concept in Diabetic Foot Management. J. Diabetes Sci. Technol..

[8] Bus S.A., Sacco I.C.N., Monteiro-Soares M., Raspovic A., Paton J., Rasmussen A., Lavery L.A., Van Netten J.J. (2023). Guidelines on the prevention of foot ulcers in persons with diabetes (IWGDF 2023 update). Diabetes Metab. Res. Rev..

[9] Arts M.L.J., de Haart M., Waaijman R., Dahmen R., Berendsen H., Nollet F., Bus S.A. (2015). Data-driven directions for effective footwear provision for the high-risk diabetic foot. Diabet. Med..

[10] Guldemond N.A., Leffers P., Schaper N.C., Sanders A.P., Nieman F., Willems P., Walenkamp G.H.I.M. (2007). The effects of insole configurations on forefoot plantar pressure and walking convenience in diabetic patients with neuropathic feet. Clin. Biomech..

[11] Mueller M.J., Lott D.J., Hastings M.K., Commean P.K., Smith K.E., Pilgram T.K. (2006). Efficacy and mechanism of orthotic devices to unload metatarsal heads in people with diabetes and a history of plantar ulcers. Phys. Ther..

[12] Chapman J.D., Preece S., Braunstein B., Höhne A., Nester C.J., Brueggemann P., Hutchins S. (2013). Effect of rocker shoe design features on forefoot plantar pressures in people with and without diabetes. Clin. Biomech..

[13] Hastings M.K., Mueller M.J., Pilgram T.K., Lott D.J., Commean P.K., Johnson J.E. (2007). Effect of Metatarsal Pad Placement on Plantar Pressure in People with Diabetes Mellitus and Peripheral Neuropathy. Foot Ankle Int..

[14] Zwaferink J.B.J., Custers W., Paardekooper I., Berendsen H.A., Bus S.A. (2021). Effect of a carbon reinforcement for maximizing shoe outsole bending stiffness on plantar pressure and walking comfort in people with diabetes at high risk of foot ulceration. Gait Posture.

[15] Preece S.J., Chapman J.D., Braunstein B., Brüggemann G., Nester C.J. (2017). Optimisation of rocker sole footwear for prevention of first plantar ulcer: Comparison of group-optimised and individually-selected footwear designs. J. Foot Ankle Res..

[16] Owings T.M., Woerner J.L., Frampton J.D., Cavanagh P.R., Botek G. (2008). Custom Therapeutic Insoles Based on Both Foot Shape and Plantar Pressure Measurement Provide Enhanced Pressure Relief. Diabetes Care.

[17] Zwaferink J.B.J., Custers W., Paardekooper I., Berendsen H.A., Bus S.A. (2020). Optimizing footwear for the diabetic foot: Data-driven custom-made footwear concepts and their effect on pressure relief to prevent diabetic foot ulceration. PLoS ONE.

[18] Chatzistergos P.E., Gatt A., Formosa C., Sinclair J.K., Chockalingam N. (2023). Effective and clinically relevant optimisation of cushioning stiffness to maximise the offloading capacity of diabetic footwear. Diabetes Res. Clin. Pract..

[19] Malki A., Baltasar Badaya M., Dekker R., Verkerke G.J., Hijmans J.M. (2024). Effects of individually optimized rocker midsoles and self-adjusting insoles on plantar pressure in persons with diabetes mellitus and loss of protective sensation. Diabetes Res. Clin. Pract..

[20] Waaijman R., Arts M.L.J., Haspels R., Busch-Westbroek T.E., Nollet F., Bus S.A. (2012). Pressure-reduction and preservation in custom-made footwear of patients with diabetes and a history of plantar ulceration. Diabet. Med..

[21] Bus S.A., Haspels R., Busch-Westbroek T.E. (2011). Evaluation and optimization of therapeutic footwear for neuropathic diabetic foot patients using in-shoe plantar pressure analysis. Diabetes Care.

[22] Paton J., Bruce G., Jones R., Stenhouse E. (2011). Effectiveness of insoles used for the prevention of ulceration in the neuropathic diabetic foot: A systematic review. J. Diabetes Complicat..

[23] Healy A., Naemi R., Chockalingam N. (2013). The effectiveness of footwear as an intervention to prevent or to reduce biomechanical risk factors associated with diabetic foot ulceration: A systematic review. J. Diabetes Complicat..

[24] Bus S.A., Waaijman R., Arts M., Haart M.D., Busch-Westbroek T., Baal J.V., Nollet F. (2013). Effect of custom-made footwear on foot ulcer recurrence in diabetes: A multicenter randomized controlled trial. Diabetes Care.

[25] Ulbrecht J.S., Hurley T., Mauger D.T., Cavanagh P.R. (2014). Prevention of recurrent foot ulcers with plantar pressure-based in-shoe orthoses: The CareFUL prevention multicenter randomized controlled trial. Diabetes Care.

[26] López-Moral M., Lázaro-Martínez J.L., García-Morales E., García-Álvarez Y., Álvaro-Afonso F.J., Molines-Barroso R.J. (2019). Clinical efficacy of therapeutic footwear with a rigid rocker sole in the prevention of recurrence in patients with diabetes mellitus and diabetic polineuropathy: A randomized clinical trial. PLoS ONE.

[27] Braun V., Clarke V. (2013). Successful Qualitative Research: A Practical Guide for Beginners.

[28] Owings T.M., Apelqvist J., Stenström A., Becker M., Bus S.A., Kalpen A., Ulbrecht J.S., Cavanagh P.R. (2009). Plantar pressures in diabetic patients with foot ulcers which have remained healed. Diabet. Med..

[29] Bus S., Mejaiti N., Dijkgraaf M. (2019). Cost-effectiveness of offloading-improved custom-made footwear to prevent plantar foot ulcer recurrence in high-risk patients with diabetes. Footwear Sci..

